# Simulating Pelvis Kinematics from Belt and Seat Loading in Frontal Car Crash Scenarios: Important Boundary Conditions that Influence the Outcome

**DOI:** 10.1007/s10439-024-03631-9

**Published:** 2024-10-28

**Authors:** Erik Brynskog, Johan Iraeus, Bengt Pipkorn, Johan Davidsson

**Affiliations:** 1 Chalmers University of Technology, Gothenburg, Sweden; 2 Autoliv Research, Vårgårda, Sweden

**Keywords:** Boundary conditions, Finite element analysis, Human body model, Pelvis, Submarining

## Abstract

**Purpose:**

The risk of submarining during automotive crashes, defined by the lap belt sliding off the pelvis to load the abdomen, is predicted to increase in future autonomous vehicles as greater variation in seating position is enabled. Biofidelic tools are required to efficiently design and evaluate new and/or improved safety systems. This study aims to evaluate the pelvis response sensitivity to variations in boundary conditions that directly influence the pelvis loads, deemed important for the submarining outcome, to facilitate a more precise comparison between finite element human body models (FE-HBMs) and post-mortem human subjects (PMHSs).

**Methods:**

A parameter study, using a one-variable-at-a-time analysis (low/high) of belt friction, seat friction, seat stiffness, and (on/off) for added belt bending stiffness, was performed using a state-of-the-art FE-HBM in four different test scenarios; one stationary, two sleds with upright occupant posture, and one sled with reclined occupant posture.

**Results:**

In the stationary scenario, both belt friction and belt bending stiffness influenced the belt folding behavior, which consequently affected the belt-to-pelvis angle at submarining. In the sled scenarios, only seat friction was found to influence the pelvis kinematics and submarining outcome, with the most biofidelic response resulting from both the low (0.2) and high (0.5) friction coefficient depending on the scenario.

**Conclusion:**

To reduce uncertainty in boundary conditions affecting the external pelvis loads and increase confidence in FE-HBM to PMHS comparisons, it is recommended that future experiments evaluate the PMHS to seat friction coefficient and that new belt modeling methods that accurately capture belt folding when interacting with soft tissues are developed.

## Introduction

*Submarining* in frontal crashes can lead to injuries related to belt-to-abdominal loading. This issue has been acknowledged since the introduction of the 3-point seat belt as standard equipment in vehicles and has been studied since the 1970s [1, 27]. Several definitions of submarining can be found in the literature, although most are variations on the classic definition presented by Adomeit et al. 1975: *“the lap belt slides over iliac crest with lap belt forces effecting the internal abdominal organs during forward displacement of the lower torso”* [1]. The submarining outcome of a specific crash is a complex loading scenario resulting from the interaction between an occupant and the restraint system. Parameters believed to contribute to occupant submarining can be categorized as belt and seat properties, constraint forces, vehicle accelerations, and occupant characteristics [24]. In a well-functioning system, the occupant kinetic energy should be absorbed by the restraint system while controlling the occupant kinematics via the strong osseous structures. During submarining, the lap belt load is suddenly transferred from the strong pelvic bone to the much softer abdominal structures which can result in injuries to the internal organs and lumbar spine. In addition, the loss of pelvis control causes the occupant to slide forward and downward, resulting in an associated risk of neck, lower thorax, and knee injuries [1].

Potential issues controlling the occupant kinematics via the hip might become further accentuated as advanced driver assistant systems (ADAS) and autonomous vehicles (AV) gain higher market shares. When vehicle occupants are less restricted by the driving task, they will gain more freedom to be involved in other activities, and as a result, desire to recline the seat to a more relaxed position has been expressed [17]. Unfortunately, traveling in a reclined position has been associated with increased mortality [7] and injury risk [26, 33] in the current vehicle fleet, however conclusions are limited by the low number of recorded reclined positions in real-world crashes.

When retrospectively analyzing real-world data to quantify the epidemiology of submarining scenarios, injuries to the abdominal organs are often used as proxy for the submarining event [20, 30]. However, “abdominal organs” are a broad category in which injuries can stem from multiple sources, not strictly related to a submarining outcome, i.e., incorrect initial belt placement or interaction with doors, the steering wheel, or other interior structures [30]. In addition, in examining the results from experiments with PMHSs, abdominal injuries have not been found despite strong indications of a submarining outcome [8, 18]. As a result, identification of submarining in real-world crashes is difficult and general claims of its prevalence are weak and possibly contradictory. A prospective approach using physical models like anthropomorphic test devices (ATDs) or numerical models like FE-HBMs can, hence, be an important complement to the retrospective analysis.

While ATDs are a common tool for vehicle safety assessment, current ATDs lack the ability to predict a submarining outcome [12], possibly due to their mechanical nature. FE-HBMs, on the other hand, have the potential to achieve a more biofidelic submarining prediction, since in addition to accommodating both a detailed anatomical representation and inclusion of population variance, they can predict kinematics and kinetics from omnidirectional loads. Multiple examples of FE-HBMs for occupant safety assessments have been published in the literature and certain contemporary publications include the Total Human Model for Safety (THUMS) [25], the Global Human Body Model Consortium (GHBMC) [9], and the SAFER HBM [29]. Relevant for this research is an enhanced version of the SAFER HBM [3] that was developed specifically to improve submarining predictability.

To render these models convincing as prospective tools for submarining evaluations, they must be validated. Since submarining is challenging to evaluate in real-world crashes, the validation would mainly relate to predicting the outcome of PMHS experiments with clear and well-documented boundary conditions. However, even these well-defined experiments are affected by uncertainties and unknowns in boundary conditions relating to the pelvis loading, which could influence the submarining outcome of the experiment. An example of such uncertainty, which is rarely estimated or reported in experimental studies, is seat and belt friction at the interaction with the PMHS. Friction is a parameter that directly relates to the constraint forces and can be influenced by factors such as type of fabric and its permeability, surface treatment, humidity, normal force magnitude, temperature, and time [6, 21, 22]. The constraint forces of the belt are also influenced by the belt kinematics, which can vary substantially from one test to another. Belt folding over the midline of the belt is one such variation which is difficult to evaluate since it is often obscured from vision, especially in submarining scenarios. Another example of uncertainty is the resulting seat stiffness in experimental setups, such as the semi-rigid seat [37], arising when different organizations make their own replica of the original design.

The aim of this study is to evaluate the pelvis response sensitivity to variations in boundary conditions that directly influence the pelvis loads and are deemed important for the submarining outcome, more specifically seat and belt friction, seat stiffness, and belt bending stiffness. Furthermore, to avoid conclusions which could be load specific, the evaluation is made for various front car crash scenarios. The study aims to present which boundary conditions that need to be prioritized in future experimental and numerical studies, to facilitate a more precise comparison between FE-HBMs and PMHSs.

## Material and Methods

An enhanced version [3] of the SAFER HBM v10 [29] was utilized in this research. Compared to the SAFER HBM v10, the enhanced model includes several updates to the hip and lower extremities made specifically to improve submarining predictability. In short, the model enhancements include

- *Skeletal updates:* Replacing the pelvis and lumbar spine with newly developed models [4, 15], validated on component level, and updating their skeletal orientation [16].
- *Soft tissue updates:* Re-meshing the fat/muscle tissue around the abdomen, hip, and thighs using updated skin geometry as the outer boundary surface. Furthermore, introducing a contact-based muscle to bone coupling at the estimated muscle insertion points of the pelvis, and a no-separation sliding contact between the fat and the abdomen muscle wall representing the fascia.
- *Joint updates:* Replacing the hip, knee, and ankle joints with kinematic joints and matching their rotational stiffness [32].

### Evaluated Experiments

Initially, a scoping review of the available biomechanical data for submarining evaluations was conducted [2]. Based on this review, a subset of four records with PMHSs targeting the average male anthropometry, including both submarining and non-submarining outcomes, were chosen and reproduced. The records comprise a stationary experiment using a belt system with rotating anchor points with three PMHSs in five different load configurations [36], a dynamic experiment on a rigid seat with nine upright PMHSs in three different seat and belt configurations [23], a dynamic experiment on a semi-rigid seat with eight upright PMHSs in two different seat and belt configurations [37], and a dynamic experiment on a semi-rigid seat with five reclined PMHSs [31]. In total, these include 27 tests in 11 different configurations with 17 submarining, nine non-submarining, and one partial submarining (one side) outcomes.

### Simulation Scenarios and Data Analysis

All simulations were performed using LS-DYNA MPP R12.2.1 (ANSYS Livermore Software Technology, California, United States) on a cluster running with 32 cores. The boundary conditions and model kinematic response were qualitatively evaluated for each simulation scenario and for all parameter settings. Selected results, deemed most relevant for the pelvis kinematic evaluation, were quantitatively evaluated by response at time of submarining, peak response, and comparison between simulated response and average response in the original experiments using the CORrelation and Analysis method (CORA v4.1.1.) [10]. The CORA score was calculated by using the default settings, i.e., equal weights assigned to corridor score and cross-correlation score, with 25% contribution from phase and size score and 50% from progression (shape) score. Furthermore, all comparisons were done with an automatically generated inner and outer corridor using the full duration of the reported time history.

**Fig. 1:**
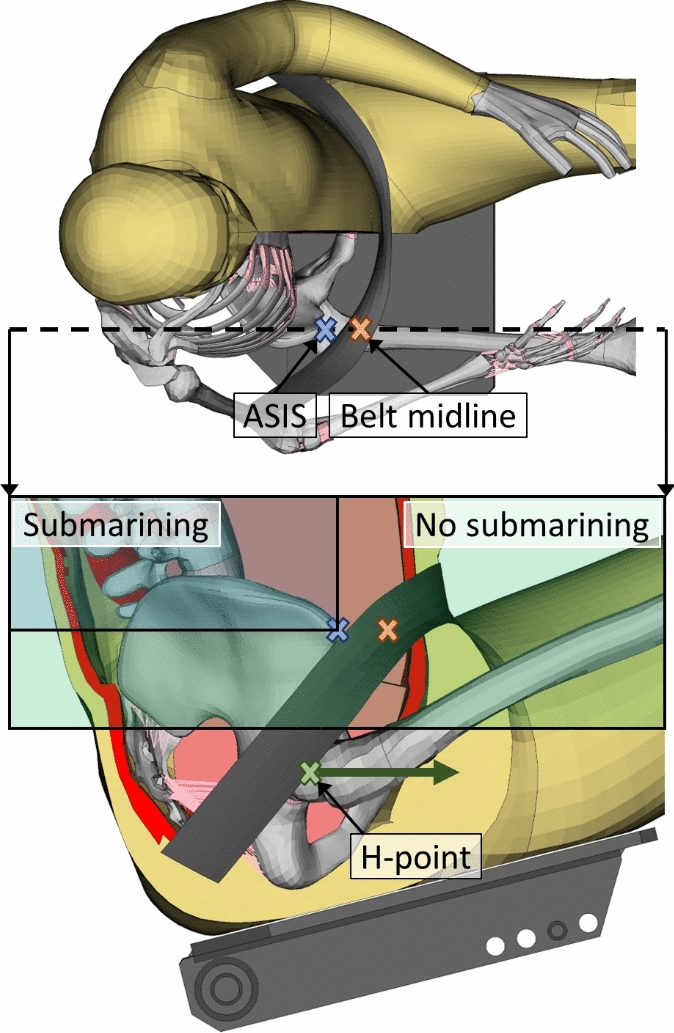
Submarining, defined as the midline of the lap belt (orange cross) moving superior and posterior to the ASIS (blue cross) in the left/right ASIS aligned sagittal planes (black dashed line for right ASIS), while the H-point (green cross) still has a forward velocity relative to the vehicle. Visually, this means that submarining occurs if the orange cross moves from the no submarining box to the submarining box, while the H-point velocity (green arrow) points forward.

Submarining was defined as the midline of the lap belt moving superior and posterior to any of the left/right anterior superior iliac spine (ASIS) in the ASIS aligned sagittal planes, while the H-point still had a forward velocity relative to the vehicle (Fig. 1). As the stationary load per definition did not have H-point velocity, the H-point velocity requirement was discarded for this scenario.

To avoid differences in submarining response due to varying modeling techniques of different experiments, an effort was made to keep methods consistent across all simulations, unless variations were explicitly specified in the records. For the baseline, this included belt modeling with six elements across the width of the belt, using quadrilateral under-integrated membrane (ELFORM = 5) elements with a thickness of 1.2 mm and a 2D seatbelt material (*MAT_SEATBELT_2D) with default settings. The loading/unloading curves for the belt material were based on a generic FE vehicle buck model [13], except in the reclined scenario where a previously validated simulation model with a detailed belt was utilized [11]. When the belt used in the experiments was defined by exact dimensions and/or force at a given strain, the mesh was made to match the reference and the material model loading curve was scaled to the given force-strain specification. If the initial slack/load, film spool effect, and load limiter behavior were not reported, these were considered as tuning parameters to match the boundary condition signals. This involved calibrating the models using the baseline configurations with the aim of matching belt and seat force signals to the experiment, before evaluating the kinematics of the model and the submarining outcome. For the added bending stiffness parameter, fully integrated membrane (ELFORM = 9) elements with a coated 2D seatbelt material (*MAT_SEATBELT_2D with FORM = − 14) were implemented [35].

**Fig. 2:**
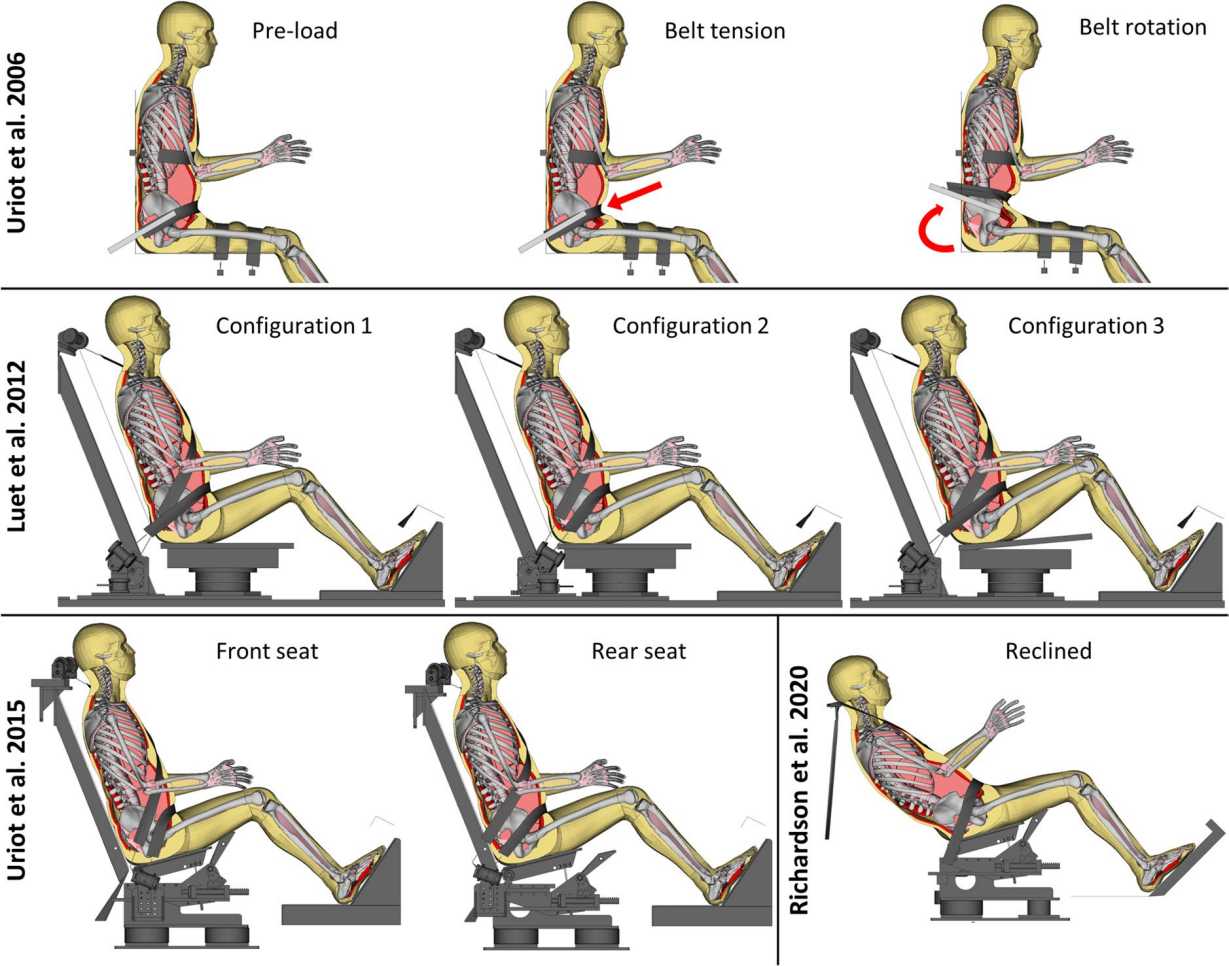
An overview of all simulation scenarios evaluated. Top row: stationary experiment with a belt system with rotating anchor points [36]. Middle row: three configurations of sled experiments [23]. Bottom row (left side): front and rear seat configurations of sled experiments [37]. Bottom row (right side): reclined sled experiments [31].

All simulations included a pre-simulation to position the SAFER HBM according to the average PMHS position reported, by prescribing nodal displacements (marionette method) on skeletal structures, see Appendix A. The re-positioned model was then de-penetrated in all contacts. To initiate the simulation scenarios, gravity was applied with global damping and constraints in the sagittal plane, such that the model reached equilibrium in 300 ms of simulation time, before ramping down the damping and applying the actual load. See Fig. 2 for an overview of all simulation scenarios.

#### Stationary Scenario [36]

**Fig. 3:**
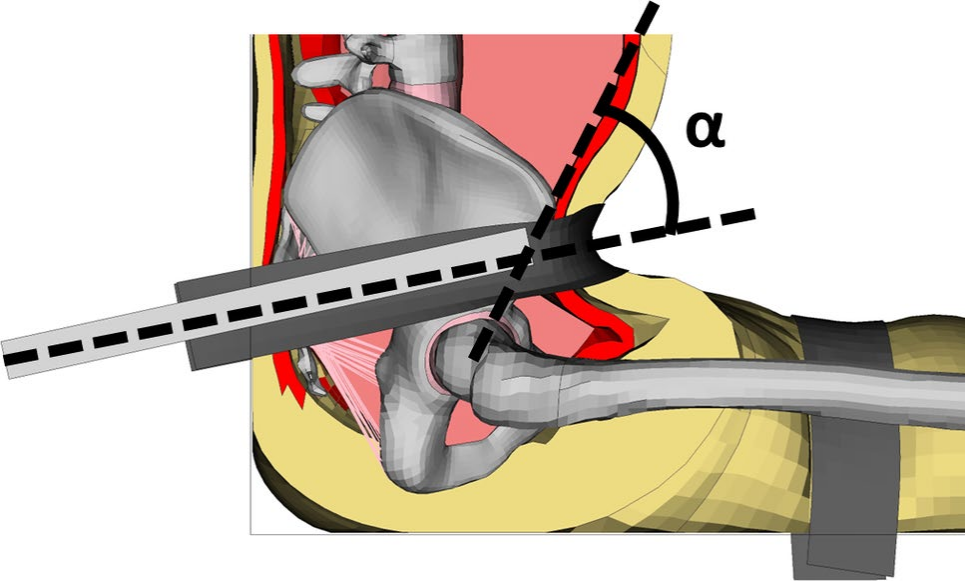
The belt-to-pelvis angle of the experiment, defined as the angle between a line connecting the belt anchors with the center of the belt and a line connecting the H-point with the ASIS in the mid-sagittal plane [36]

The reference only presents results for tests identifying a clear “un-hooking” (submarining), (i.e., test MHA 44, MHA 53, MHA 54, MHA 55, and MHA 56), hence, these particular tests were reconstructed. In the reconstruction, a rigid seat, a rigid rotating bar, a lap belt, a chest belt, and four thigh belts were simulated, see Fig. 2. The chest and thigh belts were pre-loaded at 250 N, while the ischial tuberosities were constrained in space by prescribing zero displacement. The belt was modeled at 48 mm wide and 1 mm thick with a belt stretch of 8% at a tension of 10 kN, as described in the experiments. A case specific lap belt pre-load varying from 162 to 241 N, based on recorded data when tightening the belt, was applied before the actual lap belt force curve from the experiments. When the “actual average tension” plateau was reached, the force was kept constant, while the rotating bar was spun with its “actual angular velocity,” following a specified delay between tension and rotation, based on reported data. The simulation was run until submarining occurred, at which point the belt-to-pelvis angle, defined as the angle between a line connecting the belt anchors with the center of the belt and a line connecting the H-point with the ASIS in the mid-sagittal plane (Fig. 3), was measured.

#### Upright Dynamic Scenario on Rigid Seat [23]

The reference presents three different configurations that were all reconstructed. In the reconstruction a rigid seat, a rigid footrest, a 2-point shoulder belt, and a 2-point lap belt were simulated, see Fig. 2. Lacking specific belt dimension and stiffness data from the experiments, a baseline belt (as described previously) was implemented. A retractor without force limiter was defined at each end of the lap belt and at the top end of the shoulder belt, with a belt pre-load of 50 N applied at each retractor, based on input from the study authors (Xavier Trosseille, personal communication, 2022-12-12). The belt film spool effect was used as a calibration variable to match the recorded belt force initiation.

#### Upright Dynamic Scenario on Semi-Rigid Seat [37]

The reference presents two different configurations (front/rear seat) that were both reconstructed. In the reconstruction, a semi-rigid seat, a rigid footrest, a 2-point shoulder belt, and a 2-point lap belt were simulated, see Fig. 2. The belt was modeled at 45 mm wide with a belt stretch of 9% at a tension of 10 kN, as described in the experiments. A retractor was defined at each end of the lap belt and at the top end of the shoulder belt, with a belt pre-load of 50 N applied at each retractor, based on input from the study authors (Xavier Trosseille, personal communication, 2022-12-12). The lap and shoulder belt force limiters were set to match their respective resulting average from the experiments. The belt film spool effect was used as a calibration variable to match the recorded belt force initiation.

#### Reclined Dynamic Scenario on a Semi-Rigid Seat [31]

In the reconstruction, a semi-rigid seat, a rigid footrest, and a 3-point seat integrated belt system were simulated, see Fig. 2. The simulation scenario was previously validated [11], but has been updated based on recommendations from the belt developer and to be consistent across all simulation scenarios in this study. The main updates include switching from *MAT_SEATBELT with fully integrated membrane (ELFORM = 9) elements to *MAT_SEATBELT_2D with under-integrated membrane (ELFORM = 5) elements in the baseline setup (added bending stiffness implemented as described previously) and changing the belt to HBM contact from a soft constraint formulation (SOFT = 1) to a segment-based penalty formulation (SOFT = 2).

### Parameter Variations

**Fig. 4:**
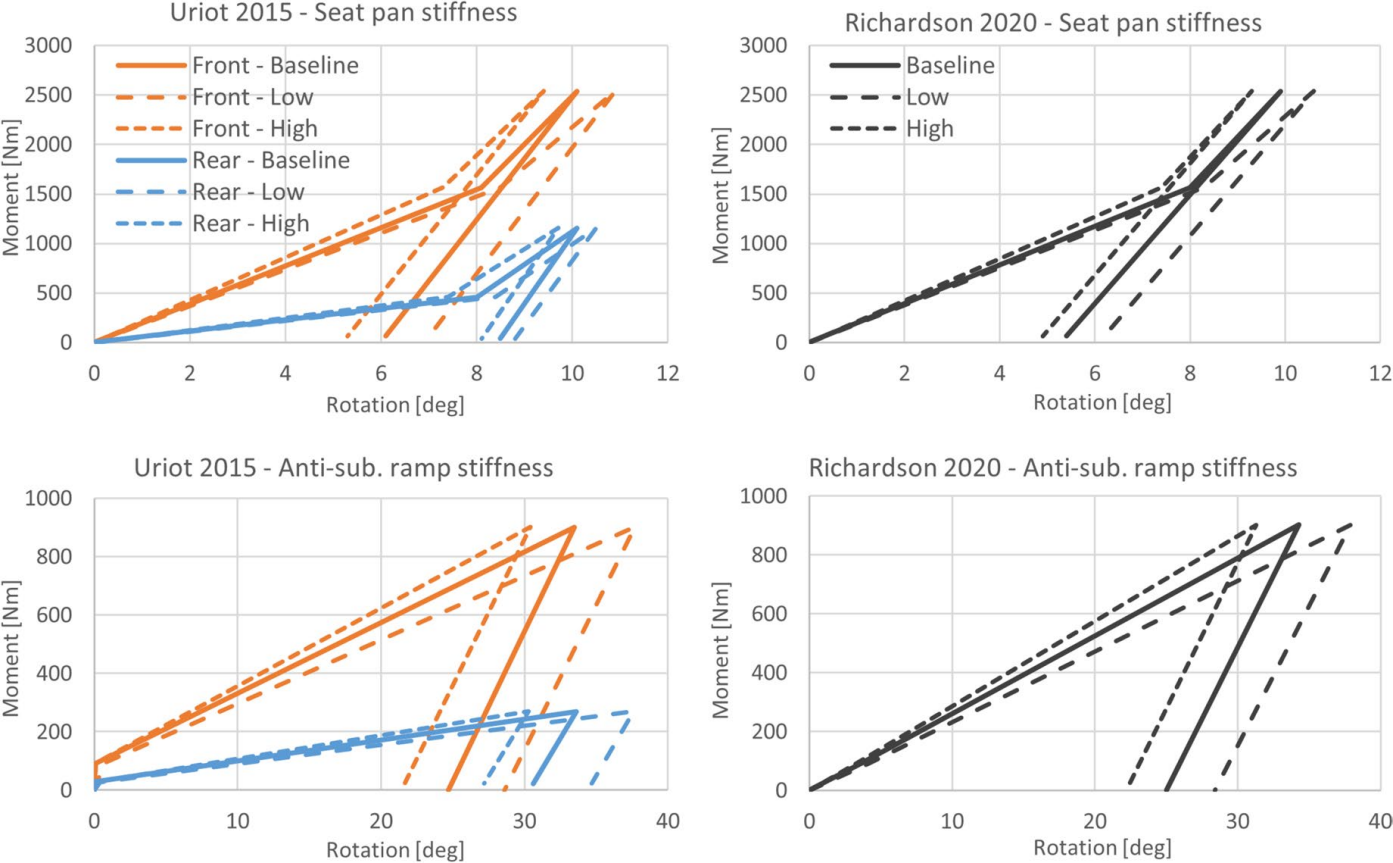
Resulting seat stiffness from parameter variation in the upright dynamic simulation scenario on a semi-rigid seat [37] (left) and the reclined simulation scenario [31] (right).

The parameter study included four factors: belt to HBM friction, seat to HBM friction, seat stiffness, and added belt bending stiffness. Each simulation scenario described above was run with a baseline parameter setting followed by a one at a time low/high level for each parameter, or on/off for the added bending stiffness, see Table 1 and Fig. 4. The belt friction parameter was included in all scenarios, seat friction parameter in the dynamic sled scenarios, seat stiffness parameter in the scenarios with a semi-rigid seat, and added belt bending stiffness parameter in all scenarios. A total of 62 simulations were performed.

**Table 1:** Parameter settings

Parameter	Baseline	Low	High
Belt friction	0.3	0.2	0.4
Seat friction	0.35	0.2	0.5
Seat stiffness	From ref.*	− 10%	+ 10%
Belt bending stiffness**	Off	On	

*Baseline seat stiffness was tuned to the reported values for the upright dynamic scenario on a semi-rigid seat [37], while the reclined scenario [31] remained as modeled in the previously validated version [11].

**Belt bending stiffness from coating on seatbelt membranes was not included as baseline (Off), or included according to [35] (On).

## Results

The evaluation of submarining or non-submarining outcome using the SAFER HBM was completed for all simulation scenarios. Of the total number of 62 simulations, 51 ran to normal termination, while 11 crashed prematurely (four in the upright scenario with a rigid seat, four in the upright scenario with a semi-rigid seat, and three in the reclined scenario). In all crashed simulations, the crash either happened after the submarining occurred or after the HBM had begun to rebound, and hence, did not affect the submarining evaluation. The crashes were always a result of a solid element with negative volume found in either the upper or the lower abdominal cavity. For a complete set of boundary condition and kinematic results for all simulation scenarios with a sled setup, presented per original study and configuration, see Appendix B.

**Fig. 5:**
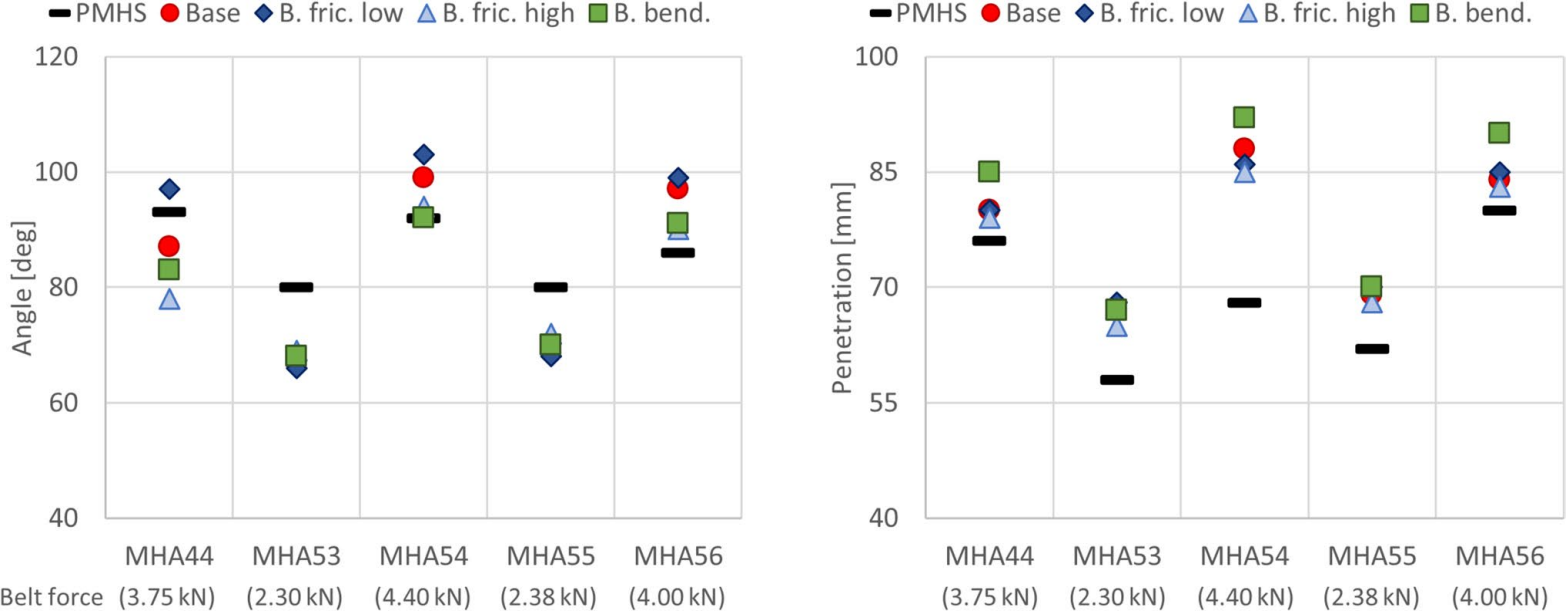
Simulated belt-to-pelvis angle at submarining (left) and umbilicus penetration at average belt tension (right) versus stationary experiments [36] (black lines). The different parameter settings are referred to as baseline (red dots), low belt friction (dark blue diamonds), high belt friction (light blue triangles), and added belt bending stiffness (green squares).

In Fig. 5, the belt-to-pelvis angles at submarining and the umbilicus penetrations at average belt tension are compared between the PMHSs from the stationary experiment [36] and the SAFER HBM, subjected to varying belt friction and a belt with/without added bending stiffness. In one case (MHA44), it was not possible to match the reported initial belt-to-pelvis angle due to anthropometric differences and possible typo in reference, see Appendix A, resulting in an initial error of 14°. In the low belt force configurations (2.30 kN for MHA53 and 2.38 kN for MHA55), the belt-to-pelvis angle was on average underestimated by − 12.5° (SD: 1.3)/− 10.0° (SD: 1.6), respectively. In the high belt force configurations (4.40 kN for MHA54 and 4.00 kN for MHA56), the belt-to-pelvis angle was on average overestimated by 5.0° (SD: 5.0)/8.3° (SD: 4.4), respectively. In the intermediate force configuration (3.75 kN for MHA44), the belt-to-pelvis angle was overestimated for low belt friction (4°) and underestimated for baseline, high belt friction, and added belt bending stiffness (−6°/− 15°/− 10°, respectively). The over-/underestimation of belt-to-pelvis angle was correlated with belt folding for all configurations, where belt folding has been defined by the mesh folding in on itself toward the belt midline, resulting in a belt width of one to five elements depending on the degree of folding, see Appendix C for example. Higher belt forces, lower friction, and added belt bending stiffness resulted in earlier and more rope-like (fewer elements across the width) belt folding with a stronger coupling to the pelvis bone. In the low belt force configurations, the belt did not fold for any of the parameter settings, resulting in limited variation for the belt-to-pelvis angle at submarining. In all simulations, the umbilicus penetration when reaching average belt tension was overpredicted with a minimum average for MHA44 (5.0 mm (SD: 2.7)) and a maximum average for MHA54 (19.8 mm (SD: 3.1)).

**Fig. 6:**
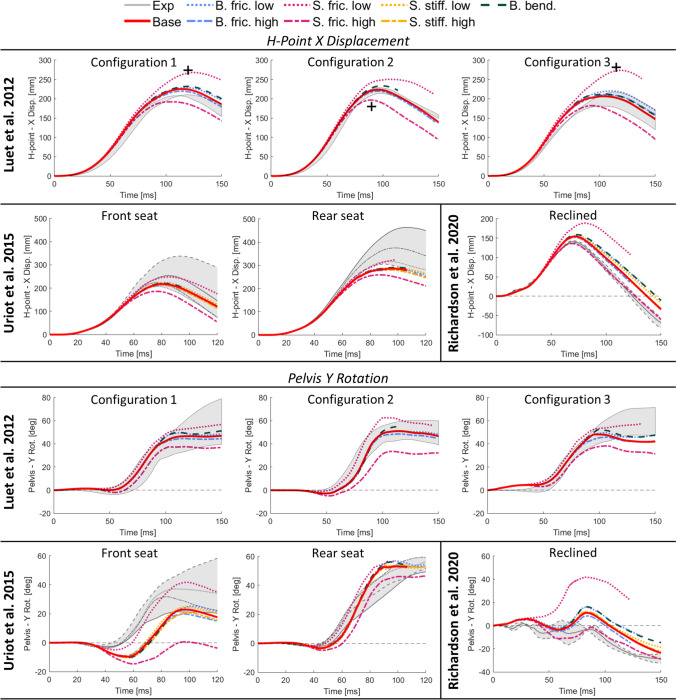
Simulated pelvis kinematics for all sled configurations versus experiments (gray envelope). The different parameter settings are referred to as baseline (red solid line), belt friction (blue), seat friction (magenta), seat stiffness (yellow), and added belt bending stiffness (dark green), with low settings as dotted lines and high settings as dashed. Top half: H-Point X displacement. Bottom half: Pelvis Y rotation. *Black crosses in top row H-point X displacements indicate peak kinematics for missing subject data (one per configuration), estimated by the current authors based on video from the experiments. These signals were not reported in the original record due to accelerometer malfunction.

In Fig. 6, the pelvis kinematics (H-Point X displacement and pelvis Y rotation) for all sled configurations and parameter settings are plotted versus the experiments. In all configurations, the seat friction variation had a clear effect on the simulated pelvis kinematics (average of absolute difference to baseline: 33.4 mm (SD: 12.4) H-point X-disp. and 13.5° (SD: 7.6) pelvis Y-rot.), while a limited influence was found for belt friction (4.6 mm (SD: 3.2) and 2.4° (SD: 1.2)), seat stiffness (3.3 mm (SD: 1.5) and 1.1° (SD: 0.8)) and added belt bending stiffness (5.8 mm (SD: 2.4) and 3.5° (SD: 1.7)). In Table 2, the CORA scores of simulated pelvis kinematics in all sled configurations versus the experimental averages are shown. Based on the CORA scores, the overall best match was found with baseline settings in the upright dynamic experiments on a rigid seat (Luet et al. 2012) [23] (average CORA = 0.93), with low (0.2) seat friction in the upright dynamic experiments on a semi-rigid seat (Uriot et al. 2015) [37] (average CORA = 0.90), and with high (0.5) seat friction in the reclined experiments (Richardson et al. 2020) [31] (average CORA = 0.88). The strongest effect of seat friction on pelvis rotation was found for the no/borderline submarining cases, i.e., configuration 2 in [23], front seat in [37], and reclined [31], where the peak pelvis rotation changed by 29°/41°/43°, respectively, when going from a low (0.2) to a high (0.5) friction coefficient, see Fig. 6. The parameter variations had limited effect on the kinetic boundary conditions, i.e., resulting belt force, seat force, foot-pan force, with the main difference stemming from seat friction on simulated seat X-force, see Appendix B. In all simulations, belt folding at varying degrees was predicted, see Appendix C for example figures.

**Table 2:**
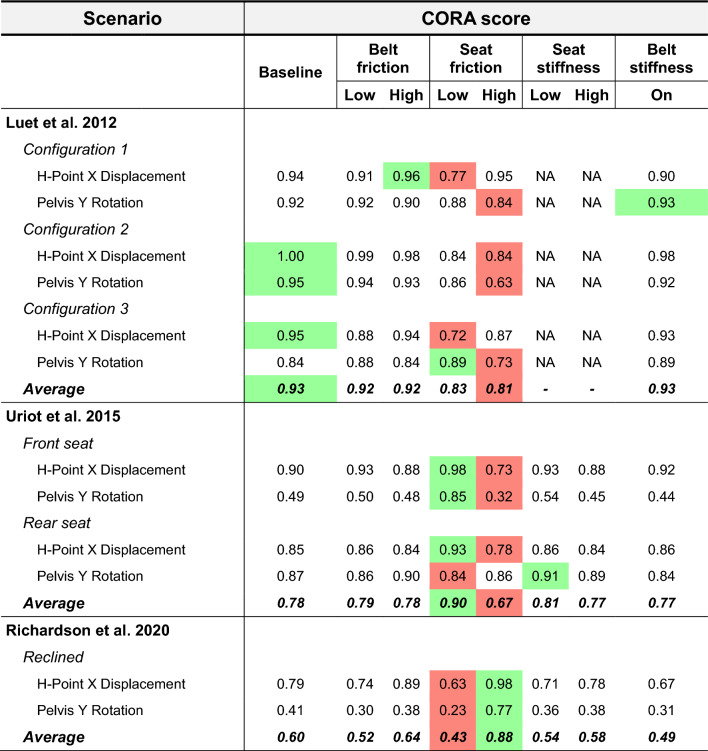
CORA scores of simulated pelvis kinematics in all sled configurations versus experimental average using default CORA settings (v4.1.1) in the full simulation time interval

A green/red box indicates the highest/lowest score for each signal

In Table 3, the submarining outcome (Yes/No as defined in Methods) of all simulation scenarios and all parameter settings are presented. A green box indicates a match with the majority outcome of the corresponding experiment, a yellow box a match with the minority outcome, and a red box not matching the outcome of any tested PMHS. For the Yes/No classification, partial submarining (only one side) has been classified as submarining. In most configurations, the submarining outcome was not affected by the parameter variations, even though a clear effect was predicted for the pelvis kinematics. Exceptions include configuration 2 in [23], where a no submarining outcome became a submarining outcome at low seat friction, and configuration 3 in [23] and rear seat in [37], where a submarining outcome became a no submarining outcome at high seat friction.

**Table 3:**
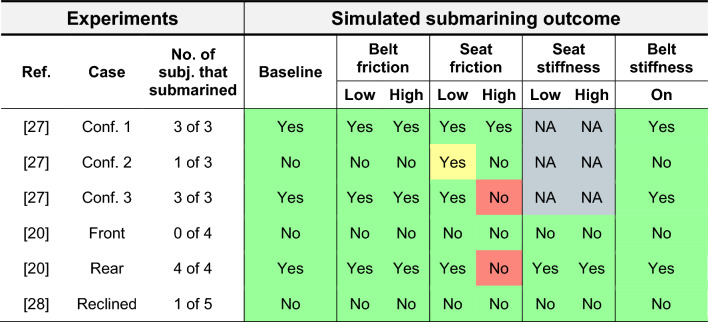
Simulated submarining outcome for all sled configurations versus experiments

A green box indicates a match with the majority outcome of the corresponding experiment, a yellow box a match with the minority outcome, and a red box not matching the outcome of any tested PMHS. For the Yes/No classification, partial submarining (only one side) has been classified as submarining

## Discussion

This paper evaluates the pelvis response sensitivity to boundary conditions that directly influence the pelvis loads, deemed important for the predicted pelvis kinematics and submarining outcome, namely belt friction, seat friction, seat stiffness, and belt bending stiffness, in multiple simulations of front car crash experiments. To the authors’ knowledge, this is the first paper to present an evaluation on the effect of these parameters in submarining scenarios.

Submarining identification can be done in many ways, i.e., lap belt force drop, belt-to-pelvis angle, hip-to-torso relative kinematics, abdominal injury pattern, pelvic strain, video analysis, etc. Most of these definitions are results from the difficult task of identifying submarining in experiments, where the pelvis is not visible in video recordings, and the belt is often obscured by the protruding abdomen at the time of submarining. This issue is highlighted in the discussion in [37] which, even though all four rear seat subjects were recorded as submarining, states that *“In fact, no resounding submarining was produced and its occurrence could even be questioned.”*. In this case, the type of abdominal injury sustained in the rear seat configuration was used to point to a submarining diagnosis. Using a signal such as abdominal injury pattern to define submarining outcome would, however, not be possible in the current version of the SAFER HBM, since the abdominal organ modeling lacks sufficient detail and corresponding injury risk functions. In simulations, the complications of submarining identification in experiments are not an issue since the exact position of all parts is known throughout the simulation. The definition used in this study, presented in Fig. 1, was selected since it matches the spirit of the classic definition [1], presented in Introduction, while avoiding false positives for submarining in the rebound phase when the belt no longer applies injury producing loads. It also allows for independent left/right side submarining identification in the respective ASIS-planes. Since submarining identification often cannot be made with the same definition for both experiment and simulation, where experiments usually utilize a combined subjective assessment of multiple signals while the simulation can utilize a strict yes/no outcome, a risk for discrepancies when comparing submarining outcomes arises. However, from Table 3, the definition presented in this paper resulted in a submarining prediction that was well aligned with the reported outcomes from the experiments.

The parameters for friction and stiffness evaluated in this study were defined as a baseline with low/high levels to capture a reasonable uncertainty interval, based on the available literature and input from relevant manufacturers in the industry. For belt friction, a baseline friction of 0.3 was selected based on seatbelt to dummy validations [11, 19], while the low (0.2) and high (0.4) levels were defined based on industry input and range used in previous studies [13]. For seat friction, a baseline friction of 0.35 was selected based on semi-rigid seat to dummy validations [11]. The low (0.2) and high (0.5) levels were selected to acknowledge the possibility of a low friction contact between fabric and steel, indicated by un-published experiments made by the Laboratory of Accidentology, Biomechanics and Human Behavior (LAB) (Jérôme Uriot, personal communication, 2024-04-04), and a high friction contact (~0.5) found between denim/sweats and a nylon covered rigid seat [34]. A wider seat friction range was further motivated by considering the unknown influence of varying fabrics used for PMHS clothing, seat surface finish, contact humidity conditions, and time on seat prior to test [6, 21, 22], and by acknowledging the large variation expected in real driving conditions when comparing, e.g., leather and fabric seats. For seat stiffness, the semi-rigid seat in the upright dynamic simulations used a baseline setting to match the reported torque-to-rotation reference [37], while the reclined semi-rigid seat simulations used the previously reported model [11], validated against a replica of the original semi-rigid seat from [37]. The low/high levels were defined as ± 10% variation in spring stiffness without further reference, see Fig. 4 for the resulting effect on seat torque-to-rotation response. The authors acknowledge that the identified effect of parameter variations is strongly related to the parameter range considered; too narrow and some of the effect could be missed, too wide and the effect could be overestimated/irrelevant. The ranges evaluated in this study were defined with the intention of capturing reasonable real-world variation present in the uncertain parameters.

In the stationary simulation scenario, belt friction had limited effect on belt-to-pelvis angle at submarining (max 2° difference from baseline) for the tests with low belt force, while a greater effect (max 7° difference from baseline) was found for the tests with high belt force. Similarly, the added belt bending stiffness resulted in max 1°/7° difference for the tests with low/high belt force when comparing with the baseline. Surprisingly, a greater underestimation of belt-to-pelvis angle (indicating earlier submarining) was associated with the low friction level in the tests with low belt force, while the same friction level was associated with a greater overestimation (indicating later submarining) in the tests with high belt force. However, analyzing the simulations, the contradictory effect appears to be explained by varying belt kinematics. In the tests with low belt force, the belt remained flat throughout the submarining event, while in the tests with high belt force the belt folded over to varying degrees. In the test with intermediate belt force, the folding/no folding outcome depended on the contact friction, where the low friction level made the belt more prone to folding and the high friction level kept the belt flat. In the scenarios with high friction, comprising a flat belt, the belt was more likely to slide on the skin surface, causing it to slide earlier than the low friction scenarios with a folded belt. In the scenarios with a folded belt, the belt became more rope-like and penetrated the soft tissue surrounding the pelvis moving it closer to the iliac spine, making it less likely to slide. Since the low friction level triggered earlier folding, the belt then had longer time to couple with the pelvis and, hence, resulting in later submarining. This explains why the greatest variation in simulated belt-to-pelvis angle at submarining was found in the test with intermediate belt force (max 19° difference between tested variables and max 10° difference to baseline). Unfortunately, it is challenging to evaluate the validity of the belt folding seen in simulations since the reproduced experiment setup included a single frontal camera, and the belt was mostly obscured by the abdomen in this view. However, the trailing edge of the belt on each side of the abdomen was visible, facilitating confirmation that the belt was not flat in any of the experiments, although the degree of folding remains unknown. Hence, the flat belt that underestimated the belt-to-pelvis angle at submarining should have folded in the simulations, likely resulting in later submarining, while the simulations with high belt force that overestimated the angle possibly should have folded to a lesser extent, likely resulting in earlier submarining. Future FE-HBM simulations would benefit from a numerically stable belt modeling technique that can accurately capture the belt folding kinematics and from PMHS testing that aims to evaluate the degree of folding seen in the experiments.

In the dynamic sled simulations, belt friction, seat stiffness, and added belt bending stiffness had limited effect on pelvis kinematics and submarining outcome. Compared to the stationary simulations, belt force was at or above the high level of ~ 4 kN for all cases. As a result, belt kinematics were more uniform across the different scenarios and always included some degree of belt folding, which could explain why belt friction was less influential than in the stationary simulations. Again, validity of the exact belt folding seen in simulations was difficult to evaluate, but varying degrees of belt folding were confirmed based on video analysis in all experiments, except in the reclined [31] where the trailing edge of the belt remained flat, while the remainder of the belt was obscured. On the other hand, seat friction influenced both pelvis kinematics and submarining outcome. In the upright dynamic rigid seat simulations representing [23], the baseline seat friction coefficient resulted in the highest CORA score when compared to the average response of the experiments. In the upright dynamic semi-rigid seat simulations representing [37], a low seat friction coefficient resulted in the highest CORA score. The main difference being a slight overestimation of pelvis rotation at the time of submarining in the rear seat, which again could be the result of excessive belt folding causing a strong coupling between the belt and pelvis, as discussed for the stationary simulations. In the reclined semi-rigid seat simulations representing [31], a high seat friction coefficient resulted in the highest CORA score. This high friction changed the pelvic rotation from a rearward rotation (iliac spine moving away from the lap belt) to a forward rotation (iliac spine moving toward the lap belt), something noted as a limitation in the previous evaluations made with the GHBMC, THUMS, and SAFER models [11]. Future FE-HBM simulations would benefit from PMHS tests that aim to evaluate the PMHS to seat friction coefficient in well-controlled representative conditions to reduce the uncertainty in boundary conditions affecting the simulated pelvis kinematics.

The simulated folding of the belt is a result of out-of-plane forces combined with the modeled belt bending stiffness, which was the specific motivation for the added belt bending stiffness parameter. However, contrary to our expectations, the belt with added bending stiffness became more sensitive to folding toward the midline than the baseline belt. Furthermore, it was more numerically unstable and failed in the buckle slipring transition for the reclined scenario (solved by including a section of the baseline belt locally over the slipring). While the coated 2D seatbelt material for added bending stiffness has been shown to capture edge folding of a belt when compressing a foam block [35], the calibration of the material parameters was done for a tension-free webbing sample bent in the longitudinal direction. This calibration is not representative of the simulated case with a stretched belt bent in both longitudinal and transverse direction. In addition, this validation was done with a much higher mesh resolution on both the soft solids (foam) and the belt (18 elements across compared to six elements in this study). As discussed in [35], the element size of the webbing mesh controls the smallest folding length, and it is possible that a higher belt mesh resolution is required to capture a more accurate folding behavior. To test this hypothesis, the upright dynamic simulation with a semi-rigid seat in the rear seat configuration [37] was rerun with 24 elements across the width of the belt (element size < 2 mm). However, the rerun results presented an even earlier folding and a premature termination of the simulation, due to a failing element in the belt webbing, see Appendix C.

To calibrate a representative seat friction coefficient to be used in simulation, when the experimental value has not been reported, it is essential to consider the complete force balance of the system. The seat friction will directly influence the seat contact force, which in turn will influence the force balance with the remaining restraint forces, e.g., belt, foot-pan, knee, etc. In the upright dynamic simulations representing [23] and [37], the main difference was found in seat X-force, while the belt force was not strongly affected (at least until the time of submarining or model rebound), see Appendix B. However, these experiments do not include foot-pan force, hence gaining a complete overview of the effect on the restraint force balance is not possible. In the reclined simulations, the effect on boundary condition forces was limited even though an effect was seen in the kinematic analysis, see Appendix B. Except for lap belt and buckle resultant force at low seat friction, the simulated boundary condition forces all stayed within the reported corridors. Therefore, based on this analysis, it is not possible to state with confidence which coefficient of friction is more representative. However, an argument for the higher coefficient could be made if one considers that the PMHSs used to construct the corridors weigh 74–75 kg, while the SAFER HBM weighs 77 kg, which should result in a slightly higher seat X-force. Given the apparent difficulty in assessing seat friction coefficient purely based on the simulated force balance, and the influence it had on pelvis kinematics and submarining outcome, the fact that the complete range of friction coefficients (0.2–0.5) could be potential candidates for a PMHS to rigid seat interaction is considered a notable limitation for FE-HBM validation. The large variation could potentially be explained by different experimental procedures at different institutes. Based on input from LAB (Jérôme Uriot, personal communication, 2024-04-04), the PMHSs were dressed in Lycra jumpsuits, the fabric to seat interaction was wet at the time of the experiment, the room was air-conditioned and not above 25°C, and the time the PMHS sat on the seat prior to the event varied extensively depending on the test [23, 37]. Based on input from University of Virginia (UVA) (Jason Kerrigan, personal communication, 2024-05-21), the PMHSs wore diapers and were wrapped in Coban before being dressed in cotton shorts, the fabric to seat interaction was dry at the time of the experiment, the room was kept at 10–12°C, and the time the PMHS sat on the seat prior to the event varied between 4 and 6 h [31]. The most notable differences are the different fabrics used to dress the PMHSs and the humidity conditions for the PMHS to seat interaction.

Due to limitations in simulation resources, this study did not perform a full-factorial evaluation of the included parameters. This would have required 300 simulations compared to the 62 simulations performed. Due to this limitation, it is not possible to draw conclusions on potential interaction effects that could exist in the parameter space. In addition, the study only evaluated an enhanced version of the male SAFER HBM [3], consequently the results could be both model and sex specific, as anatomical differences [5] could affect the lap belt interaction. The authors, hence, recommend future work to evaluate the included parameters with other FE-HBMs to establish if the conclusions can be stated as general. The use of a 50%ile male HBM (stature 1.75 m, weight 77 kg) also explains some of the discrepancies seen between the simulated response and the experimental results. For example, in the upright dynamic experiments with a rigid seat [23], the average PMHS had a stature of 1.66 m and weight of 63 kg which explains the higher shoulder belt force and seat X-force found in simulation. In the upright dynamic experiments with a semi-rigid seat [37], the average PMHS had a stature of 1.71 m and a weight of 72 kg. In this scenario, the shoulder belt included a force limiter which enabled matching between simulated and experimental responses; however, the resulting belt pay-out was greater in the simulations which, again, can partly be explained by the taller and heavier model. Another factor influencing torso kinematics is the lack of simulated rib fractures where the experiments on average produced 32 [23], 23 [37], and 10 [31] total rib fractures. Instead of simulating rib fracture, the SAFER HBM uses an injury risk function to estimate the number of fractures based on local strain measures. While the model’s ribcage has been thoroughly validated [14, 28], it has not been validated for the extensive fractures seen in these experiments. The effect on pelvis kinematics due to differences in upper torso kinematics is unknown and should be treated as a limitation when evaluating the parameter effect.

In conclusion, for the parameter variations evaluated in this study, belt friction and added belt bending stiffness had a limited effect on pelvis kinematics and submarining outcome in the simulated dynamic scenarios (average of absolute difference from baseline: < 6 mm H-point X-disp. and < 4° pelvis Y-rot.) but could influence belt folding, as seen in the simulated stationary scenarios, which in turn affects the submarining timing. Seat friction had a more distinct effect on both pelvis kinematics and submarining outcome (33 mm and 13.5°), while seat stiffness had no notable effect (3 mm and 1°). With unknown boundary conditions that substantially influence the simulated response, model validation becomes challenging and the results are uncertain. In addition, there is an added risk of tuning the boundary conditions until a satisfactory model response is achieved. However, if the tuned condition is not representative to the real condition, this results in false model validation since the response is calibrated based on the specific settings of the simulation. To reduce the uncertainty in boundary conditions affecting the external pelvis loads and facilitate a more precise comparison between FE-HBMs and PMHSs, it is recommended that future experiments should evaluate the PMHS to seat friction coefficient in well-controlled representative conditions. Ideally, this should be done prior to testing each PMHS; however, since this might not always be feasible, an isolated experiment could be considered if a similar testing protocol is followed. In addition, it is recommended that future work targets a numerically stable belt modeling technique that can accurately capture the belt folding kinematics as a stretched belt is engaging with a soft, adipose-like structure, while being transversely compressed toward the midline.

## Figures and Tables

**Fig. 7:**
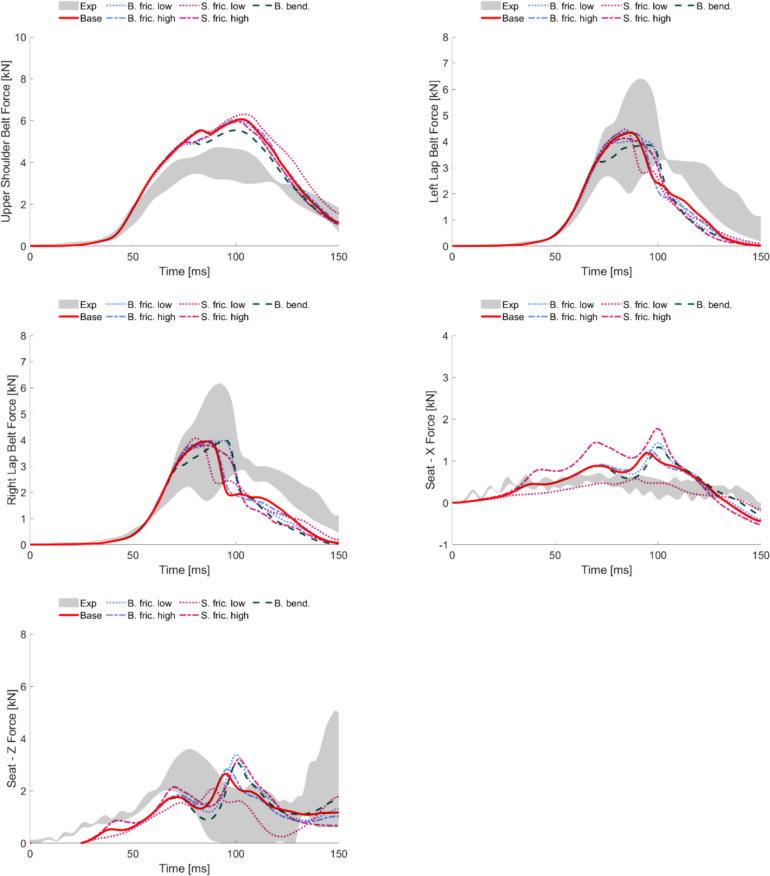
Boundary condition signals for the SAFER HBM compared to the response envelope in Configuration 1 [23]. The different parameter settings are referred to as baseline (red solid line), belt friction (blue), seat friction (magenta), and added belt bending stiffness (dark green), with low settings as dotted lines and high settings as dashed

**Fig. 8:**
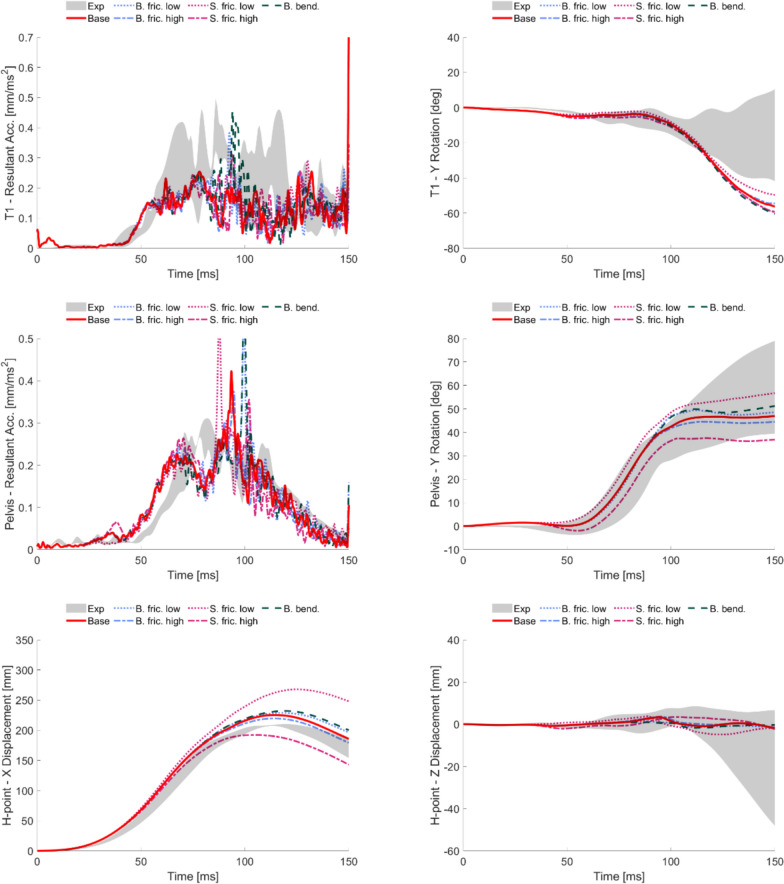
Kinematic signals for the SAFER HBM compared to the response envelope in Configuration 1 [23]. The different parameter settings are referred to as baseline (red solid line), belt friction (blue), seat friction (magenta), and added belt bending stiffness (dark green), with low settings as dotted lines and high settings as dashed

**Fig. 9:**
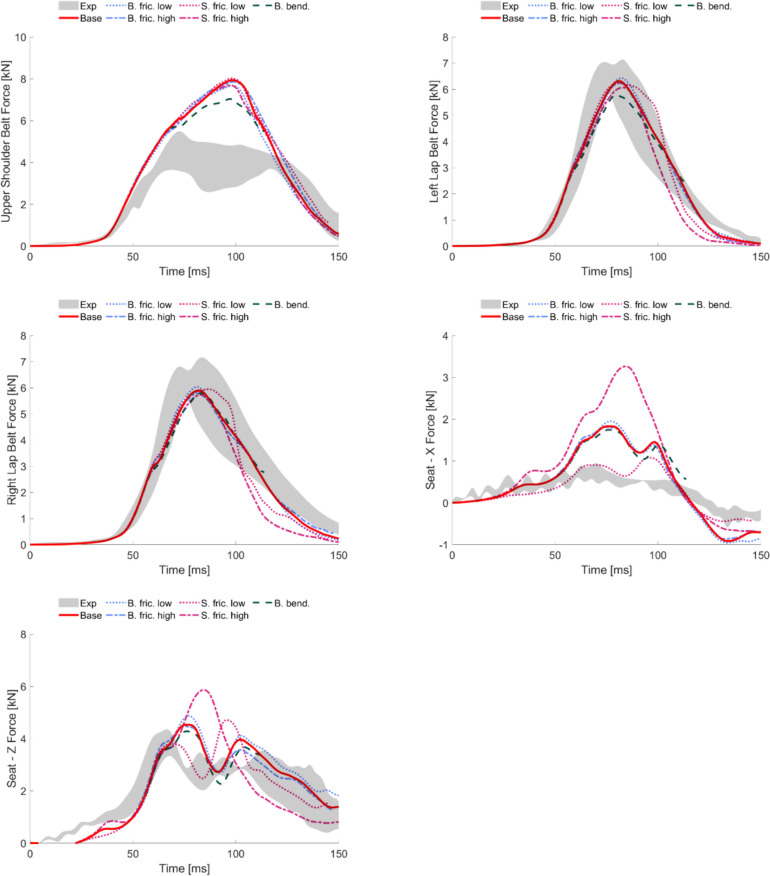
Boundary condition signals for the SAFER HBM compared to the response envelope in Configuration 2 [23]. The different parameter settings are referred to as baseline (red solid line), belt friction (blue), seat friction (magenta), and added belt bending stiffness (dark green), with low settings as dotted lines and high settings as dashed

**Fig. 10:**
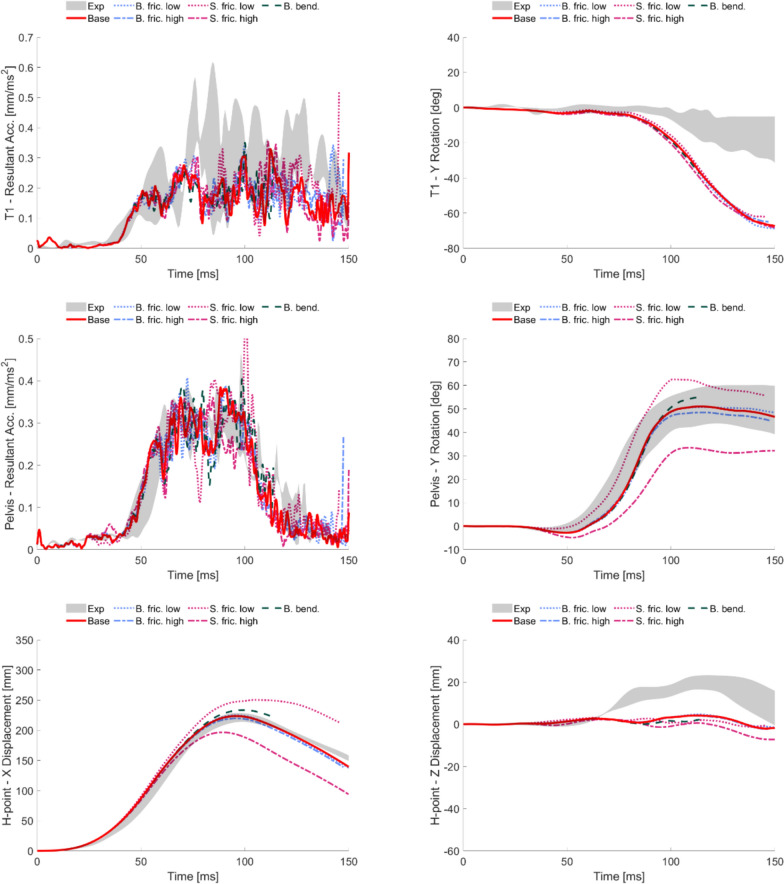
Kinematic signals for the SAFER HBM compared to the response envelope in Configuration 2 [23]. The different parameter settings are referred to as baseline (red solid line), belt friction (blue), seat friction (magenta), and added belt bending stiffness (dark green), with low settings as dotted lines and high settings as dashed

**Fig. 11:**
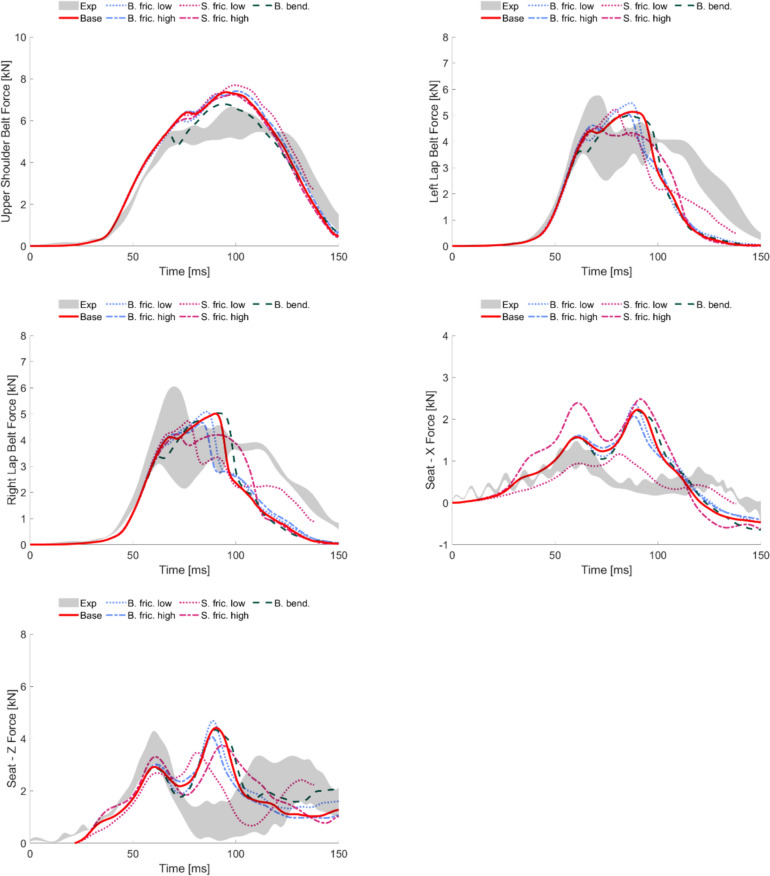
Boundary condition signals for the SAFER HBM compared to the response envelope in Configuration 3 [23]. The different parameter settings are referred to as baseline (red solid line), belt friction (blue), seat friction (magenta), and added belt bending stiffness (dark green), with low settings as dotted lines and high settings as dashed

**Fig. 12:**
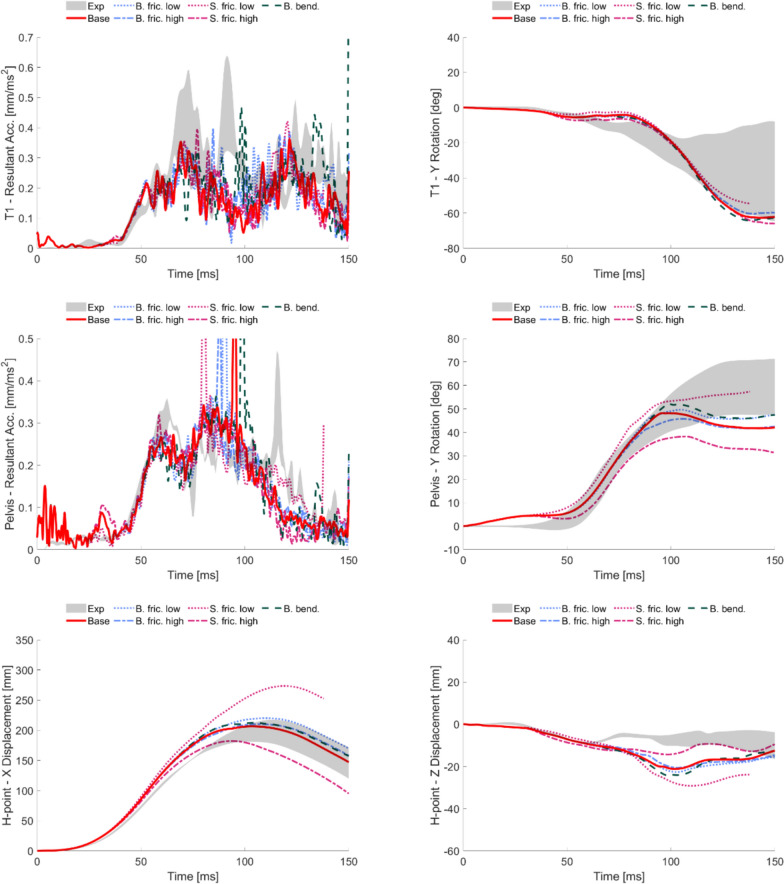
Kinematic signals for the SAFER HBM compared to the response envelope in Configuration 3 [23]. The different parameter settings are referred to as baseline (red solid line), belt friction (blue), seat friction (magenta), and added belt bending stiffness (dark green), with low settings as dotted lines and high settings as dashed

**Fig. 13:**
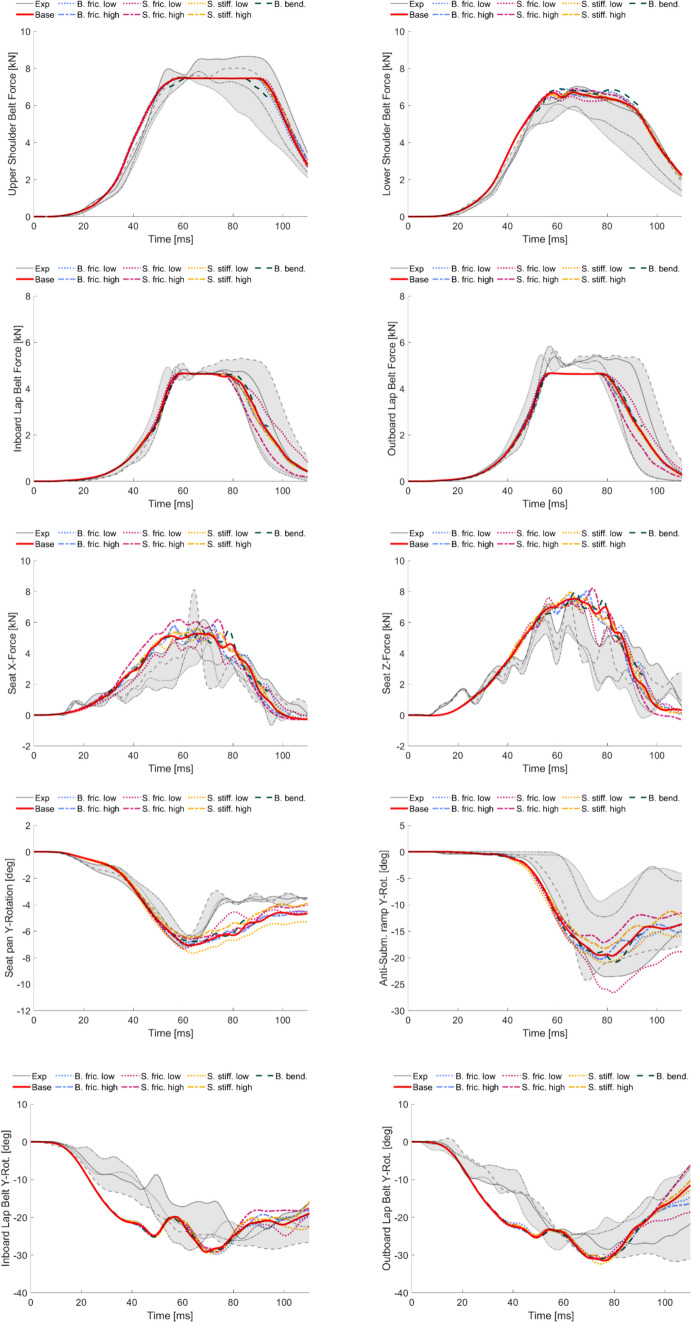
Boundary condition signals for the SAFER HBM compared to the subject response in the front seat configuration [37]. The different parameter settings are referred to as baseline (red solid line), belt friction (blue), seat friction (magenta), seat stiffness (yellow), and added belt bending stiffness (dark green), with low settings as dotted lines and high settings as dashed

**Fig. 14:**
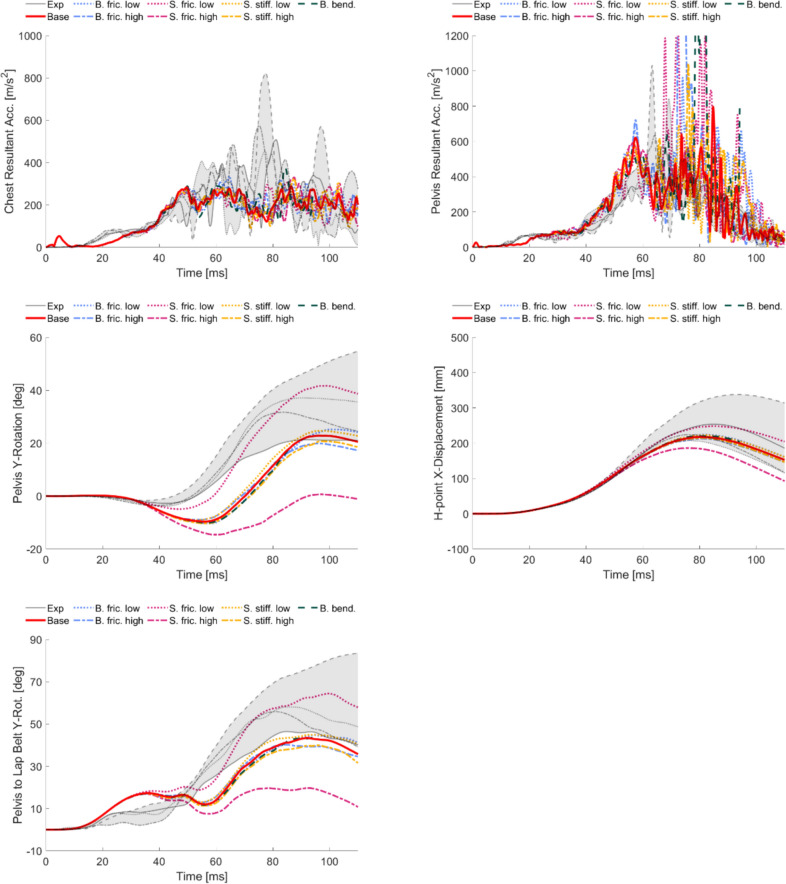
Kinematic signals for the SAFER HBM compared to the subject response in the front seat configuration [37]. The different parameter settings are referred to as baseline (red solid line), belt friction (blue), seat friction (magenta), seat stiffness (yellow), and added belt bending stiffness (dark green), with low settings as dotted lines and high settings as dashed

**Fig. 15:**
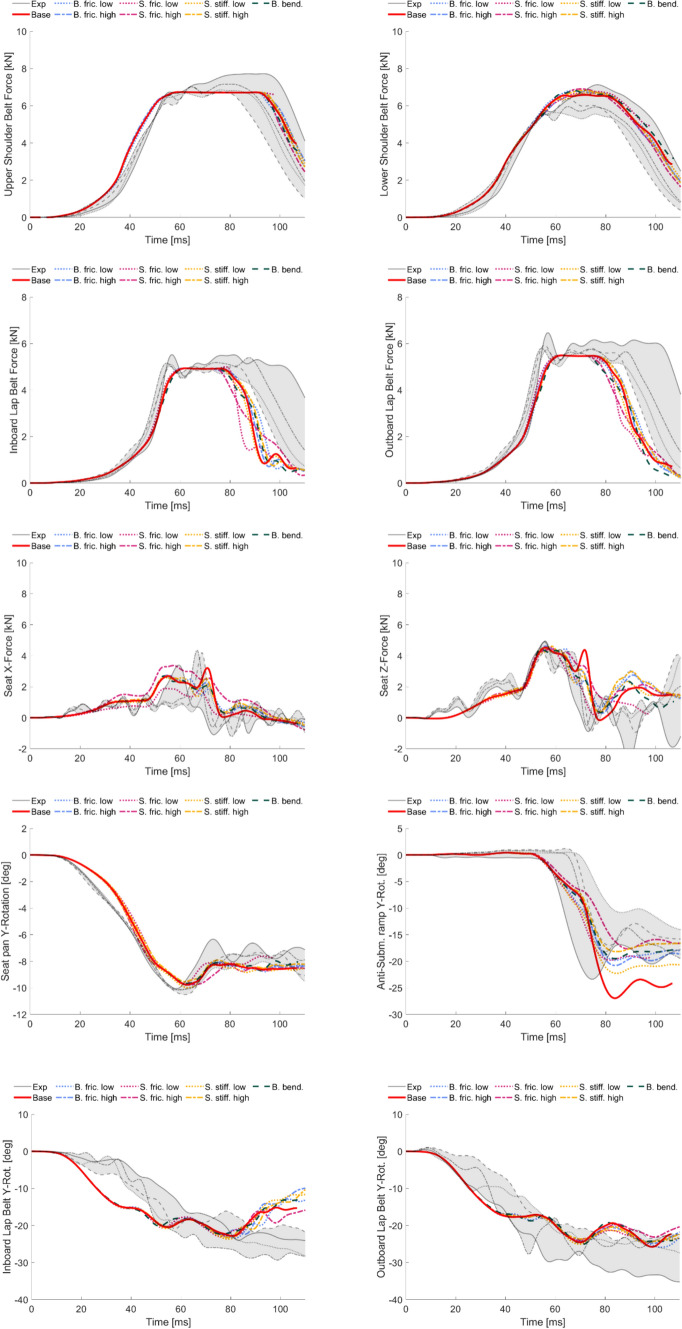
Boundary condition signals for the SAFER HBM compared to the subject response in the rear seat configuration [37]. The different parameter settings are referred to as baseline (red solid line), belt friction (blue), seat friction (magenta), seat stiffness (yellow), and added belt bending stiffness (dark green), with low settings as dotted lines and high settings as dashed

**Fig. 16:**
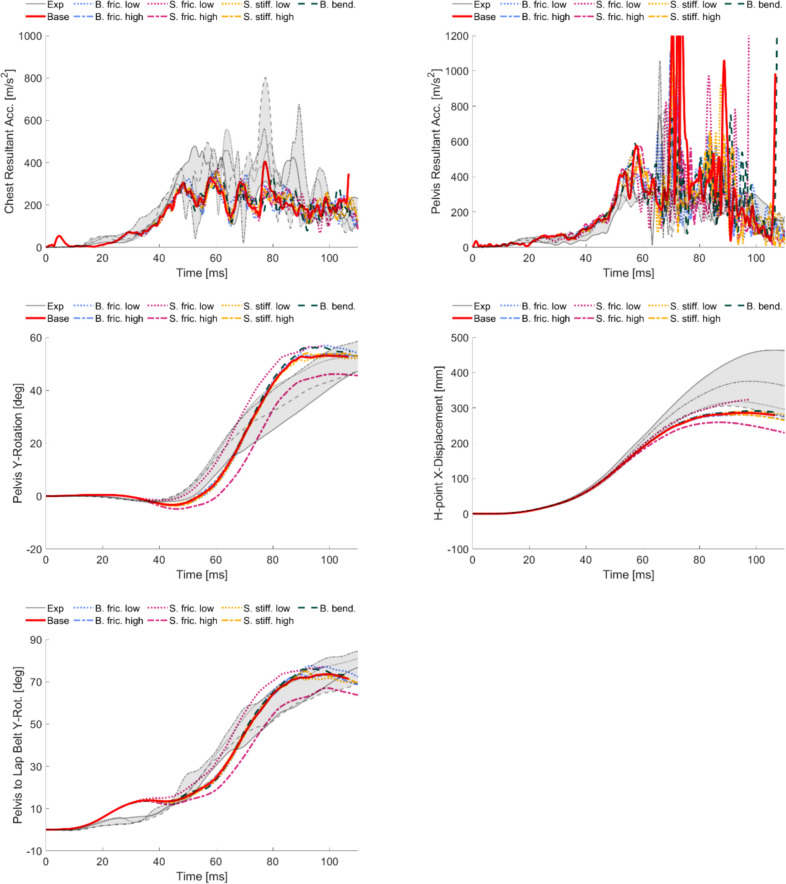
Kinematic signals for the SAFER HBM compared to the subject response in the rear seat configuration [37]. The different parameter settings are referred to as baseline (red solid line), belt friction (blue), seat friction (magenta), seat stiffness (yellow), and added belt bending stiffness (dark green), with low settings as dotted lines and high settings as dashed

**Fig. 17:**
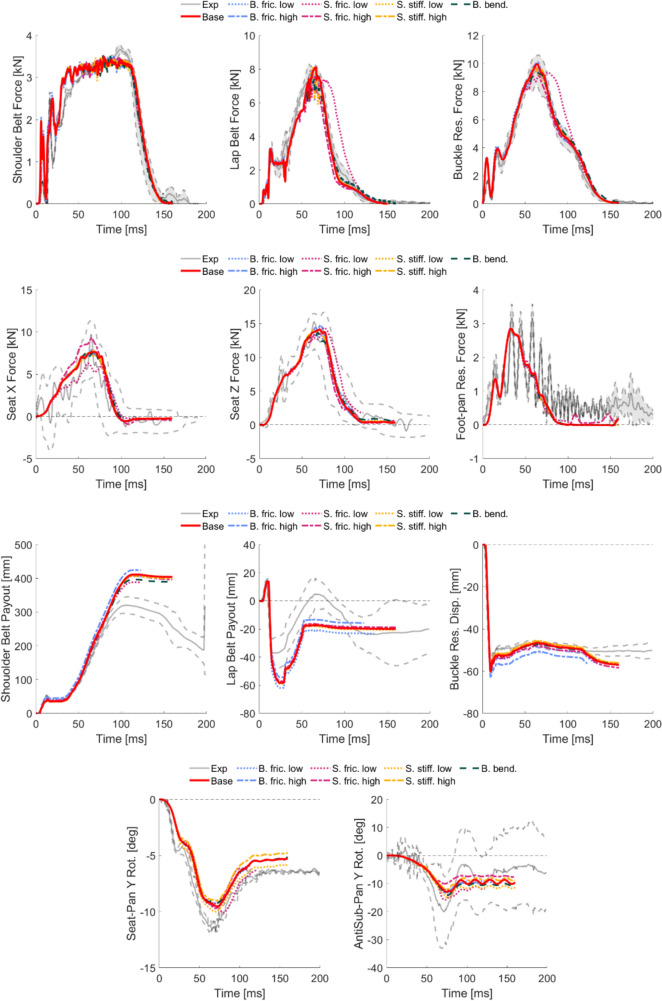
Boundary condition signals for the SAFER HBM compared to the subject response in the reclined configuration [31]. The different parameter settings are referred to as baseline (red solid line), belt friction (blue), seat friction (magenta), seat stiffness (yellow), and added belt bending stiffness (dark green), with low settings as dotted lines and high settings as dashed

**Fig. 18:**
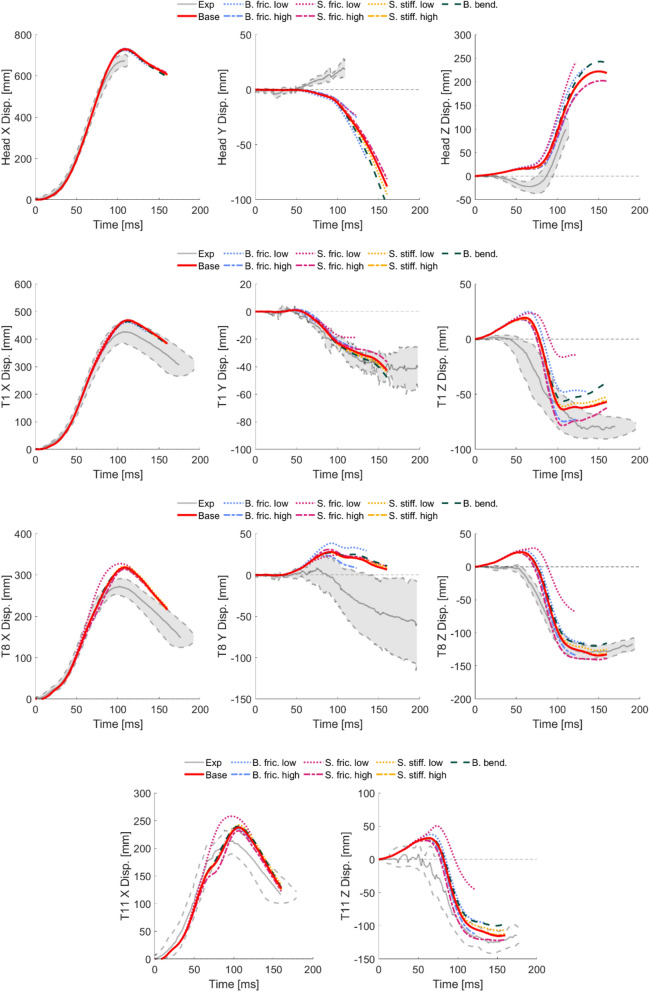

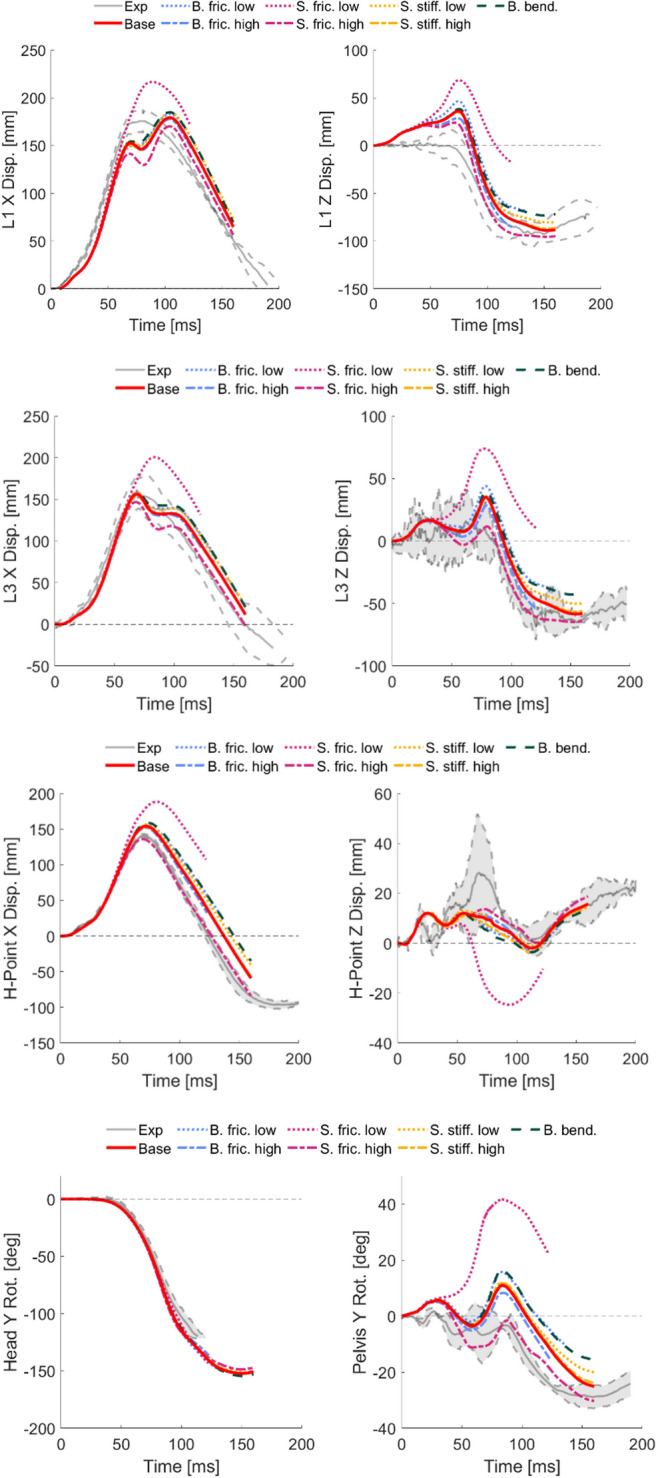
Kinematic signals for the SAFER HBM compared to the subject response in the reclined configuration [31]. The different parameter settings are referred to as baseline (red solid line), belt friction (blue), seat friction (magenta), seat stiffness (yellow), and added belt bending stiffness (dark green), with low settings as dotted lines and high settings as dashed

**Fig. 19:**
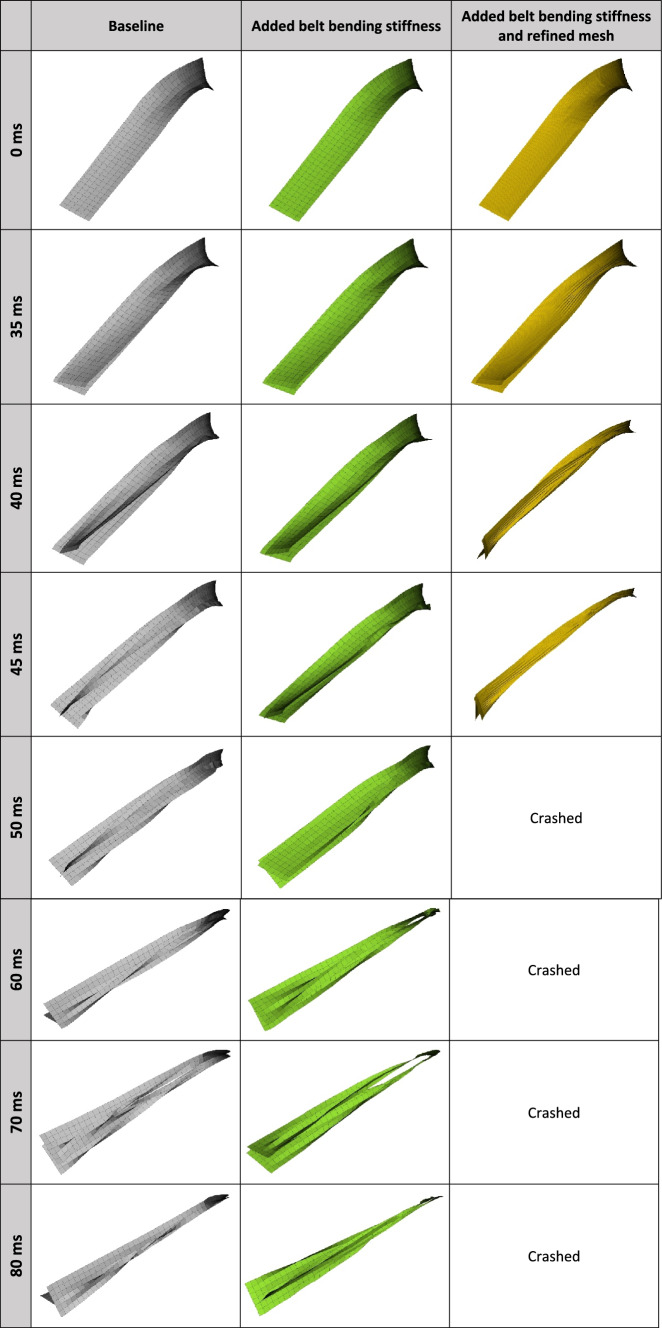
Belt kinematics in upright dynamic scenario on a semi-rigid seat in the rear seat configuration. For the baseline and added belt bending stiffness case, the folding initiates at ~ 40 ms and then gradually progresses until only one element width of belt remains. For the refined mesh, the folding starts earlier, at ~ 35 ms, followed by a quick collapse toward the centerline of the belt before the simulation crashes at ~ 45 ms

## Appendix A

Resulting initial position for all simulation scenarios compared to reported initial position per original study and configuration.

### Stationary Scenario [36]

See Table

**Table 4:** Initial angle between belt and pelvis, defined as the angle between a vector connecting the H-point and the ASIS and a vector connecting the belt anchor point and the center of the lap belt, all projected on the mid-sagittal plane

	Initial angle (deg)				
Test number	MHA44*	MHA53	MHA54	MHA55	MHA56
Experiment	43	29	30	31	31
Simulation	29	30	30	31	31
Difference	14	1	0	0	0

The angle is presented for each reconstructed PMHS test and for the corresponding simulation case. *Initial angle of test MHA44 could not be replicated, possibly due to two reasons. Firstly, the PMHS was lighter (64 kg) than the SAFER HBM (77 kg) and had thinner thighs as a result. Secondly, when reanalyzing the data from the experiment, the resulting angle was measured as 34°. It is, hence, possible that the reported angle is a typo

4.

### Upright Dynamic Scenario on Rigid Seat [23]

See Table

**Table 5:** Average PMHS position in all configurations by [23] compared to the SAFER HBM position at the onset of acceleration, i.e., post-settling with gravity

	H-point		Pelvis	Femur	Tibia	Lap belt
	*X* (mm)	*Z* (mm)	(deg)	(deg)	(deg)	(deg)
*Configuration 1*						
#631	90.2	− 85.4	60	27	52.5	36.5
#632	78.3	− 84.5	58	22.1	51.5	40
#633	47.8	− 76.3	76	13.4	39	35
Average exp.	72.1	− 82.1	64.7	20.8	47.7	37.2
Simulation	72.2	− 93.9	64.0	19.5	50.8	39.9
Difference	0.1	− 11.8	− 0.7	− 1.3	3.1	2.7
*Configuration 2*						
#636	78.1	− 80.6	73	27	53.5	49
#635	81.4	− 78.9	68	12.7	35.3	48
#634	81.1	− 84.7	73	20.8	45.8	49.5
Average exp.	80.2	− 81.4	71.3	20.2	44.9	48.8
Simulation	80.1	− 91.2	73.7	20.1	52.2	51.2
Difference	− 0.1	− 9.8	2.4	− 0.1	7.3	2.4
*Configuration 3*						
#639	100.4	− 96.7	56	25.2	55.4	40
#638	88.2	− 88.3	67	30	57	35
#637	72.6	− 81	47	24.4	52.5	34
Average exp.	87.1	− 88.7	56.7	26.5	55.0	36.3
Simulation	87.3	− 98.6	54.9	25.4	60.1	39.9
Difference	0.2	− 9.9	− 1.8	− 1.1	5.1	3.6

A positive *X* difference indicates that the model sat in front of the PMHS average position, a positive *Z* difference indicates that the model sat below the PMHS average position, and a positive angle is defined around the global *Y*-axis pointing from left to right

5.

### Upright Dynamic Scenario on Semi-Rigid Seat [37]

See Table

**Table 6:** Average PMHS position in all configurations by [37] compared to the SAFER HBM position at the onset of acceleration, i.e., post-settling with gravity

	H-point		Pelvis		Lap belt	
	*X* (mm)	*Z* (mm)	PI (deg)	HI (deg)	Out (deg)	In (deg)
*Front seat*						
BIO22	58	− 135	112	72	64	59
BIO23	40	− 132	125	77	69	66
BIO24	30	− 127	109	68	64	64
BIO25	41	− 134	125	77	67	65
Average exp.	42.3	− 132.0	117.8	73.5	66.0	63.5
Simulation	41.7	− 139.2	116.0	76.6	72.5	72.2
Difference	− 0.6	− 7.2	− 1.8	3.1	6.5	8.7
*Rear seat*						
BIO26	75	− 149	132	82	53	41
BIO27	40	− 123	115	76	51	49
BIO28	48	− 134	109	64	51	51
BIO29	42	− 129	107	59	48	46
Average exp.	51.3	− 133.8	115.8	70.3	50.8	46.8
Simulation	50.7	− 140.9	116.0	76.6	52.1	51.9
Difference	− 0.6	− 7.1	1.2	6.3	1.3	5.1

A positive *X* difference indicates that the model sat in front of the PMHS average position, a positive *Z* difference indicates that the model sat below the PMHS average position, and a positive angle is defined around the global *Y*-axis pointing from left to right

6.

### Reclined Scenario on a Semi-Rigid Seat [31]

See Table

**Table 7:** Average PMHS position in reclined configuration [31] compared to the SAFER HBM position at the onset of acceleration, i.e., post-settling with gravity

	Average exp.			Simulation			*Difference*		
Measurement point	*X* (mm)	*Z* (mm)	Angle (deg)	*X* (mm)	*Z* (mm)	Angle (deg)	*X* (mm)	*Z* (mm)	Angle (deg)
Head origin position	3512.5	1137.6		3512.7	1124.3		0.2	13.3	
Head angle			33.6			33.6			0
T1 origin position	3467.3	996.3		3467.2	975.8		− 0.1	20.5	
T8 origin position	3421.4	844.3		3421.9	836.5		0.5	7.8	
T11 origin position	3374.2	787.5		3362.1	784.5		− 12.1	3	
L1 origin position	3320.9	744.1		3310.3	746.2		− 10.6	− 2.1	
L3 origin position	3255.0	701.7		3251.6	711.6		− 3.4	− 9.9	
Pelvis origin position	3191.4	577.0		3192.6	587.5		1.2	− 10.5	
Pelvis angle (PS to ASIS w.r.t. vertical)			74.3			76.4			2.1
Pelvis angle (PSIS to ASIS w.r.t. horizontal)			63.3			63.6			0.3
Right knee position	2658.7	750.9		2667.9	752		9.2	− 1.1	
Left knee position	2660.2	754.1		2667.9	752		7.7	2.1	
Right heel position	2419.7	350.9		2419.4	349.5		− 0.3	1.4	
Left heel position	2418.8	351.2		2419.4	349.5		0.6	1.7	
Sternum	3299.3	928.8		3300.9	926.5		1.6	2.3	
H-point center	3077.4	643.3		3077.5	648.5		0.1	− 5.2	
*Vertebrae bodies with respect to horizontal*									
T1			0.0			0.6			0.6
T8			36.0			33.4			− 2.6
T11			49.0			49.7			0.7
L1			57.0			56.4			− 0.6
L3			55.0			52.5			− 2.5

A positive *X* difference indicates that the model sat in front of the PMHS average position, a positive *Z* difference indicates that the model sat below the PMHS average position, and a positive angle is defined around the global *Y*-axis pointing from left to right

7.

## Appendix B

Complete set of results for all simulation scenarios with a sled setup, presented per original study and configuration.

### Upright Dynamic Scenario on Rigid Seat—Configuration 1 [23]

See Figs. 7 and 8.

### Upright Dynamic Scenario on Rigid Seat—Configuration 2 [23]

See Figs. 9 and 10.

### Upright Dynamic Scenario on Rigid Seat—Configuration 3 [23]

See Figs. 11 and 12.

### Upright Dynamic Scenario on Semi-Rigid Seat–Front Seat [37]

See Figs. 13 and 14.

### Upright Dynamic Scenario on Semi-Rigid Seat–Rear Seat [37]

See Figs. 15 and 16.

### Reclined Scenario on a Semi-rigid Seat [31]

See Figs. 17 and 18.

## Appendix C

Example figures of belt folding in a scenario with extensive folding. The example comes from the upright dynamic scenario on a semi-rigid seat in the rear seat configuration. The left column shows the baseline belt (*MAT_SEATBELT_2D, FORM = 0, ELFORM = 5, 6 elements across width), the middle column shows the added belt bending stiffness parameter (*MAT_SEATBELT_2D, FORM = − 14, ELFORM = 9, 6 elements across width), and the right column shows the same as the middle column but with a refined mesh (24 elements across width). The baseline and the added belt bending stiffness both ran to normal termination, while the refined mesh crashed in a collapsing belt element at ~ 45 ms (Fig. 19).
